# How Clinically Efficient Is Lumacaftor/Ivacaftor for Cystic Fibrosis Patients? An Updated Literature Review

**DOI:** 10.7759/cureus.12251

**Published:** 2020-12-24

**Authors:** Sumera Perveen, Muhammad Reza Chaudhry, Sarah AlBabtain, Sana Amreen, Simrandeep K Brar, Mehwish Zeb, Safeera Khan

**Affiliations:** 1 Internal Medicine/Family Medicine, California Institute of Behavioral Neurosciences & Psychology, Fairfield, USA; 2 Family Medicine, Ibne Sina Hospital Parco Mid-Country Refinery, Muzaffargarh, PAK; 3 Psychiatry, California Institute of Behavioral Neurosciences & Psychology, Fairfield, USA; 4 Public Health and Preventive Medicine, St. George's University School of Medicine, St. George's, GRD; 5 Psychiatry and Behavioral Sciences, California Institute of Behavioral Neurosciences & Psychology, Fairfield, USA; 6 Internal Medicine, California Institute of Behavioral Neurosciences & Psychology, Fairfield, USA; 7 Internal Medicine/Pediatrics, California Institute of Behavioral Neurosciences & Psychology, Fairfield, USA; 8 Pediatrics, Khyber Teaching Hospital, Peshawar, PAK

**Keywords:** cystic fibrosis, cystic fibrosis/therapy, cystic fibrosis/drug therapy, lumacaftor, ivacaftor/lumacaftor combination, ivacaftor, orkambi

## Abstract

Cystic fibrosis (CF) is an autosomal recessive illness caused by the defective cystic fibrosis transmembrane conductance regulator (CFTR) gene. These patients suffer from repeated chronic sinuses and lung infections, resulting in frequent hospital admissions and antibiotic (Abx) courses. These are the major contributing factors responsible for a low health-related quality of life (HRQoL) and increasing the disease burden. The introduction and approval of CFTR modulators-lumacaftor (LUM) and ivacaftor (IVA) in 2015 by the US Food and Drug Administration (FDA) reduced the mortality and morbidity rates associated with the disease. In 2018, the FDA approved these drugs from age two and five years with two copies of F5806 del. This literature review aims to present the studies centered on the clinical effects of LUM/IVA. We searched for the relevant articles, from 2016 to 2020, in PubMed Central (PMC), Google Scholars, and Journal of Cystic Fibrosis. LUM/IVA has a broader range of effects. They showed marked improvement in the reduction of pulmonary exacerbations (PEx), Hospitalization rates, Abx use, and modification in forced expiratory volume in one second (FEV1) status of pre-existing severe lung disease. Now, there is a need for an initiative to conduct more clinical trials and studies in the future to assess and evaluate the long-term clinical benefits and safety of LUM/IVA therapy in all age groups.

## Introduction and background

Cystic fibrosis (CF) is a chronic, progressive, and genetic disease affecting more than 90,000 people worldwide. There are approximately 1000 new cases diagnosed each year. The primary defect is the mutant cystic fibrosis transmembrane conductance regulator (CFTR) gene, which codes for CFTR protein, either absent or defective [1-2]. Defective CFTR protein acts as abnormal channels present on the body's mucous membranes as in the respiratory, gastrointestinal, and reproductive systems. These protein channels are responsible for chloride and fluid movement from cells of specific regions. The regular activity of the channel is halted by mutations either caused by protein production or processing, gating or, conduction abnormalities [3,4]. The results are the thick and sticky secretions instead of thin, normal secretions in targeted regions that are pathognomonic of these patients' signs and symptoms.

This disease shows its clinical impact as early as in the newborns and becomes a progressive disease from childhood to adulthood. It is a long-term and life-shortening disease affecting the health-related quality of life (HRQoL). The patients presented with the repeated chest infections, pulmonary exacerbations (PEx), decreased basal metabolic index (BMI), nutritional deficiencies, social life impact, and fertility/pregnancy issues [3].

The treatment was initially considered a supportive therapy in terms of antibiotic, anti-inflammatory, and rehabilitation before developing CFTR modulators in the last two decades. The main target of CFTR modulators is the CFTR protein mutation-expressions. There are >19,000 of these mutations; the most common transformation is F508 del, with an allelic frequency of 75%. It presents in at least one copy in nearby 90% of patients in which CFTR protein cannot make its standard 3D shape [1]. It becomes a misfolded protein exposed to endoplasmic reticulum-associated degradation (ERAD) and unable to carry out average conductance of chloride ions due to trafficking defects as well [1,4]. The gating mutation 3 is G551D in which the CFTR channels display regular cell surface expression with gates unable to open, and thus, the normal chloride movement is interrupted through this [1].

The importance of early initiation of CFTR modulators in patients can be measurable with the overall first benefits of treatment on sufferers. These modulators will be a miracle drug, lessen their agony, improve their quality of life, decrease mortality, morbidity [5,6], and repeat hospitalization [7]. Adherence to therapy has additional beneficial effects as compared to non-adherence patients. The full results give some comfort and relief to patients and caregivers as it minimizes the social burden.

**Figure 1:**
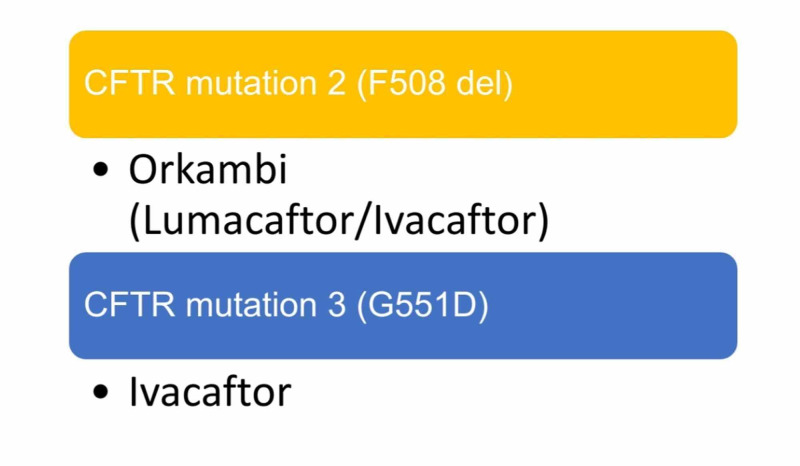
Mutations and specific drugs CFTR: cystic fibrosis transmembrane conductance regulator

The specifically approved drug targeting the mutation 2 (F508 del) is the combination of lumacaftor (LUM), the corrector, and ivacaftor (IVA), the potentiator, named Orkambi [1,8-11]. IVA alone is a drug of choice for the defective gating channel mutation 3 (G551D mutation) [1]. Figure 1 illustrates mutations and specific medications.

This review article will sum up and give readers the overall clinical effects of LUM and IVA on the patients under one umbrella. It also covers the clinical outcome associated with HRQoL, PEx frequency and pulmonary function, pathogen acquisition, pregnancy, macrophage function, physical activity, and exercise tolerance, along with hospitalization rate.

## Review

Methods

We searched comprehensively using PMC, Google Scholars, and Journal of the European Cystic Fibrosis Society (Journal of Cystic Fibrosis) with the help of the following keywords/medical subject headings (MeSH) terms, both alone or in combination, cystic fibrosis, cystic fibrosis/therapy, cystic fibrosis/drug therapy, lumacaftor, ivacaftor/lumacaftor combination, ivacaftor, Orkambi. The initial search generated 80 studies; we shortlisted only 64 relevant studies after removing the irrelevant and duplicate tasks. Finally, we included 38 studies for the review after eliminating the 26 tasks in a final scan. Quality assessment wasn't done. 

Inclusion/exclusion criteria

**Figure 2:**
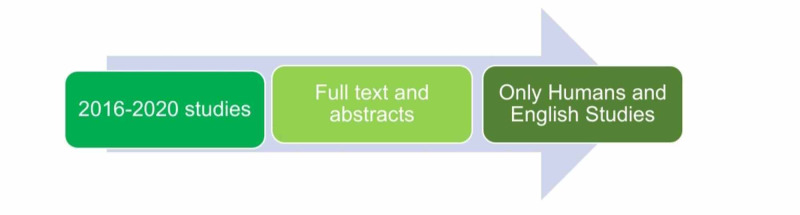
Collaborative study summary representation

We selected studies related to our research question and chosen the research papers published in the English language from 2016 to 2020 for our review, including full texts and abstracts. We had studies of all types and designs from all geographical areas and included only human studies. Figure 2 shows the collaborative study summary representation.

Discussion

Effects of Lumacaftor and Ivacaftor in Lung Functions and Pathogen Acquisition

CF disease-causing defective epithelial ion transport in an airway tract causes trapping of mucus inside the lung's lining responsible for the chronic inflammation and easy nidus for bacteria to attack as there is a faulty airway defense mechanism. The overall cascade results in repeated lung infection/ PEx in both the pediatric group and adult. All the above process is the main reason for the compromised and damaged lungs in this disease. These patients seek medical attention repeatedly and need intravenous or either inhalation Abx to overcome their respiratory deterioration.

Changes in airway microanatomy and PEx

LUM and IVA combination have a tremendous role in lung disease as they enhance the CFTR epithelial ion transport, increased ciliary beat, clearance with increased airway surface liquid penetration, and decrease mucous viscosity. Birket et al. conducted an in-vitro study in 2016 and found that IVA had displayed augmented airway ciliary transportation in F508del CFTR with reduced mucous active viscosity [1]. These improvements at the microanatomical levels in vitro showed improved lung function activity and ion transport as intensified sweat chloride variations. LUM restored the partial action of the F505del mutant to some extent when used alone [1]. However, the beneficial effects are obtained with IVA's addition in the treatment, reflected as patients' improved spirometry findings.

PEx is the causative factor for increased mortality and morbidity in patients in all age groups. With advancements in the medical field, LUM and IVA's introduction means being the patients' real-life. In 2019, McColley et al. designed a randomized control trial (RCT). They used the post hoc analysis of pooled phase 3 data (NCT01807923, NCT01807949) and, after screening and randomization, included 1108 patients who received one of the study drugs [12]. The trial's primary purpose was to compare the rates of PEx in the LUM/IVA group vs. placebo group based on ppFEV1 (percent predicted forced expiratory volume in one second). The conclusion was LUM treated patients (n=369) demonstrated considerably fewer PEx rates, and for those with LUM/IVA, the PEx rate was 0.60/patient/year. They noted the above findings for even patients with no early improvement in lung function. The extension of TRAFFIC AND TRANSPORT was carried out as PROGRESS in RCT of 2017, by Konstan et al. They enrolled 1030 patients in PROGRESS. They assessed the safety of LUM/IVA in a long-term fashion with the findings of a decreasing trend of PEx, for a 12-month duration with LUM/IVA. The same safety picture of LUM/IVA was constant to the previous RCT. They also observed the overall benefit of continued treatment for a long duration [13]. The non-adherence to treatment resulted in increased PEx frequency, as shown by a study exhibited on a Medicaid Population of one state by Tessel et al. in 2019. The 21 patients in the trial showed no decreased PEx rates after LUM/IVA therapy initiation as the study group left the treatment due to many safety concerns. They also concluded that LUM/IVA treatment reduced the Hospitalization rate along with the first visit to the emergency room [7]. The LUM/IVA adherence resulted in improved lung disease, as one of the observational studies by Diab-Caceres et al. in 2018 on 20 patients, reflecting the enhanced lung function in patients who were therapy adherent over a long period [14]. Hassan et al., in their retrospective cohort study in 2016, studied the full picture of IVA effects on PEx, Hospitalization, and Abx use. The study displayed the reduced rates of PEx and declined rates in other areas of study interest in IVA-treated cases [15].

Changes in FEV1

The patients exhibited a decreased FEV1 as the illness progresses with chronic inflammation, additional PEx rates, and, eventually, lung tissue damage. Although the baseline FEV1 is essential to reflect the clinical effect of LUM/IVA. As to how low or better the value of FEV1 at the start of LUM/IVA therapy depicted the treatment response and how much further decline can be expected in the patients by the researchers. The better the FEV1 value in the patients means the reserve capacity of lung function, the best will be the treatment response seen in early weeks to months of the start of therapy with LUM/IVA. This effect was demonstrated in a clinical trial in 2017 by Labaste et al. The study included 32 pediatric patients, and the conclusion was that the sufferers with advanced lung disease and low FEV1 at the time of drug therapy were at more risk for a further decline in FEV1 [16]. Another clinical trial by Burgel et al. in 2020 enrolled 845 patients. This clinical trial assessed and compared the effects of LUM/IVA based on FEV1 values. They established that group less than 40 FEV1 showed more discontinuation rates of therapy than other groups. They also found reduced Abx use and increased BMI in all three groups [17].

**Figure 3:**
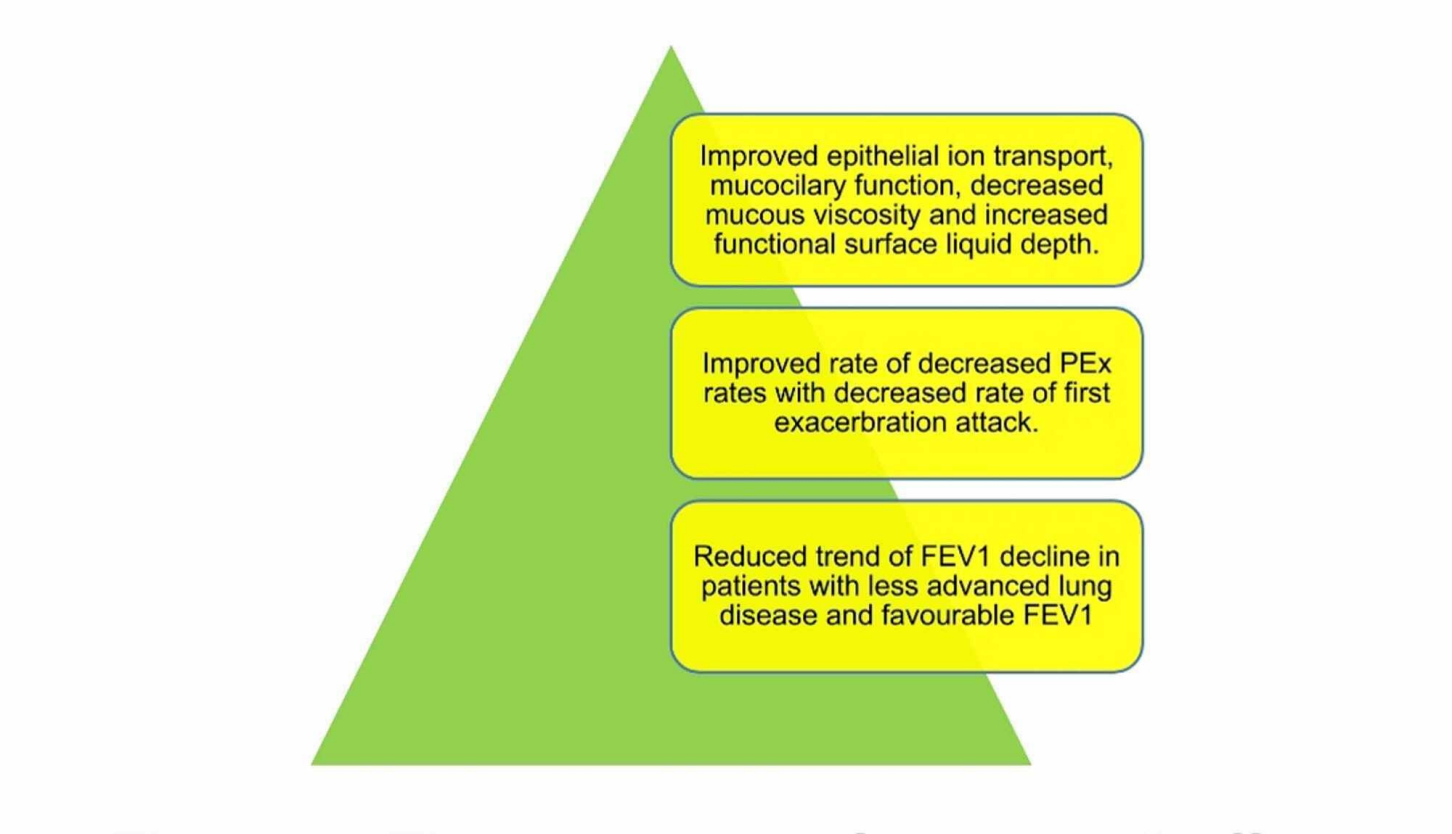
The overall outline of the drug's effect on lung PEx: pulmonary exacerbation; FEV1: forced expiratory volume in one second

Tong et al. estimated the clinical efficacy and safety of LUM/IVA in a case-control study of the 12-month duration. They conducted this study on 105 patients and revealed the decreased trend of FEV1 along with reduced PEx rates together with a declined rate of first PEx episode, which required Abx [18]. A retrospective study by Grady et al. in 2019 also supported the research mentioned above. The study group's 15 patients showed a lower rate in FEV1 after therapy with LUM/IVA [19]. Figure 3 shows the overall outline of the drug's effect on the lung.

Changes in macrophages and bacterial acquisition

LUM/IVA has a wide range of coverage over CF monocyte-derived macrophages (MDMs). As defective CFTR gene generated faulty and reduced CFTR over MDMs, it weakens in enhanced apoptosis and reduced phagocytic activity [20]. An experimental study in 2018 by Zhang et al. evaluated the effects of both LUM/IVA over the macrophages. They observed that IVA had a remarkable impact on macrophage's primary phagocytosis compared to LUM/IVA [20]. A clinical trial by Barnbey et al. in 2018 also evaluated the effects of LUM and IVA in macrophage phagocytic activity. The study of 20 enrolled patients (13 CF patients and 07 healthy patients with wild type CFTR) depicted LUM's enhanced effects over macrophage-phagocytic action against Pseudomonas aeruginosa (PA). However, IVA reduced this effect of LUM if given in combination. This study's primary result was an improved macrophage ability to kill PA and increased the chances of reduced Hospitalization and Abx use [21].

Lower respiratory tract infections with PA are more commonly observed among these patients. Abx is necessary to clear these infections to increase their survival and improve the quality of life they are spending. In a cohort study in 2019, Singh et al. assessed the outcome of LUM/IVA over getting the new bacterial infection of PA (methicillin-resistant *S. aureus* (MRSA) or methicillin-resistant *S. aureus* (MSSA)) in two time period (2008-2011, Era1) and (2012-2015.Era 2) [22]. They included 193 patients, showed the essential findings of delayed new bacterial acquirement with P=0.008 in ERA2 vs. ERA 1. The group without treatment with LUM/IVA reflected the slower trend of bacterial acquisition with no statistical findings (P=0.10) [22].

IVA alone or with LUM (Orkambi) acts synergistically with polymyxin B. An in vitro-study by Schneider et al. in 2016 revealed these three drugs did not affect PA infection if used alone. However, it showed favorable results as a 100-fold decrease in overall bacterial load or count when used in combinations [23]. These findings were even persistent after 24 hours of observation as well. It is a perfect combination for the patients who have been fighting against their chronic lung infections for many years.

Another impressive effect of Orkambi is the repair of primary lung epithelium. An in-vitro study was published in 2018 over the airway cultures. Adam et al. estimated the influence of Orkambi over the repairing of lung epithelium. They exhibited that only the combination named Orkambi showed a substantial repairing effect over the lung epithelium even in the presence of PA infections [24]. The results of the reviewed studies are summarized in Table 1.

**Table 1:** Summary of studies covering lung effects and bacterial infection RCT: randomized control trial; LUM: lumacaftor; IVA: ivacaftor; CF: cystic fibrosis; PEx: pulmonary exacerbation; Abx: antibiotic; I.V: intravenous; ppFEV1: percent predicted forced expiratory volume in one second; FEV1: forced expiratory volume in one second; PA: *Pseudomonas aeruginosa*; MIC: minimum inhibitory concentration; BMI: basal metabolic index; COMP: comparator

Author Name	Year of Publication	Type of Study	Number of Patients	Purpose of the Study	Results/Conclusions
McColley et al. [12]	2019	RCT	1108	To evaluate and compare the effects of LUM/IVA in the treated group on PEx (rates, severity) and FEV1 changes vs. a placebo group.	This study observed fewer PEx in LUM/IVA treated patients than a placebo group with a 0.60 rate of PEx/person/year.
Konstan et al. [13]	2017	RCT	1030	To assess the efficacy and safety of LUM and IVA.	They observed an overall reduction in PEx and FEVI decline rates in patients who continued the treatment.
Burgel et al. [9]	2020	Observational study	845	This study assessed the safety and efficacy of LUM/IVA in adolescents and adults.	They found significant progress in nutritional status and lung disease in therapy tolerant patients than those who discontinued the treatment.
Burgel et al. [17]	2020	Clinical trial	845	Evaluated the effects of LUM/IVA in patients with ppFEV1 was less than 40 and greater than 90 with those patients with ppFEV1 40-90 over the first year of drug initiation.	The group with ppFEV1 <40 showed higher (2.8%) discontinuation rates than other groups. They exhibited a decreased rate of I.V Abx use in all three subgroups along with increasing BMI.
Singh et al. [22]	2019	Cohort study	193	Assessed the impact of LUM and IVA on patients for new bacterial acquisition.	They displayed that those patients receiving LUM/IVA showed a significant statistical delay of new bacterial acquisition in Era2 vs. Era 1(P=0.008). Patients without these drugs showed a slower tendency of recent bacterial addition in Era2 vs. Era 1 with no statistically significant. (P=0.10)
Tong et al. [18]	2020	Case-control study	105	Estimated the rate of PEx requiring I.V ABx, mean change in FEV1, and adverse effects of LUM/IVA over the 12-months.	They observed reduced PEx and a decline rate of FEV1 and extended time for the first exacerbation. The adverse effects of treatment were too frequent and responsible for the high discontinuation ratio.
Hassan et al. [15]	2016	Retrospective cohort study	84	Assessed the changes in PEx rates, Hospitalization, and Abx use in patients initiated on IVA treatment during 12- month duration.	There was a noteworthy reduction observed in PEx rates, Hospitalization, and inhaled Abx use in patients who initiated IVA.
Labaste et al. [16]	2017	Clinical trial	32	Measured an acute FEV1 change in pediatric patients with the first dose of LUM/IVA.	Patients with previously severe airway disease and already low FEV1 were at more risk for the reduction of FEV1.
Schneider et al. [23]	2016	In vitro study	22	They studied the combined effects of IVA, LUM/IVA with polymyxin B. They also assessed their role against polymyxin resistant bacteria (MIC>4mg/L).	The study concluded the synergistic effects of IVA and LUM/IVA with polymyxin B against bacterial infections.
Tesell et al. [7]	2019	Observational study	21	Compared the PEx rates before the start of LUM/IVA in the Medicaid population.	No apparent difference in PEx rates after drug initiation in the Medicaid population was observed by them as there was a high frequency of low adherence to treatment.
Diab-Caceres et al. [14]	2018	Retrospective observational study	20	Evaluated the effects of LUM/IVA in patients (age six years and above).	The study reflected the improvement in severe PEx episodes with fewer Hospitalization with no changes in BMI and FEV1 seen. With treatment, the number of Abx use days reduced from the previous 14.9 days to 5.8 days.
Barnaby et al. [21]	2018	In vitro study	20	The impact of LUM alone or with IVA on macrophage- phagocytic ability against PA was evaluated.	CF-Macrophages showed a better ability to phagocyte and kill P.A with LUM alone as compared to IVA. Orkambi or IVA alone shown low pro-inflammatory cytokines release.
Zhang et al. [20]	2018	Experimental study	-	The differential effects of IVA and LUM/IVA on macrophage functions dependent on the patient's genotype were studied by them.	General enhancement of macrophage functions observed in IVA-treated CF patients in the context of progress in macrophage-mediated bacterial killing resulted in an 89% significant reduction in PA load in IVA-treated patients as compared to LUM/IVA with a slight decrease.
Feng et al. [2]	2018	Cohort study	143	Estimated the effectiveness of IVA on hospitalization rate a year before and after drug initiation.	A reduction of 55% in Hospitalization rate was observed by the study at 0.57 admission/person-year before filling IVA medicine to 0.26 after a year of serving treatments for adults and children.
Grady et al. [19]	2019	Retrospective study	15	Estimated the relation between ppFEV1 and the number of inducing factors.	This study showed a statistically significant inverse relationship between ppFEV1 and baseline ppFEV1 with no relation with BMI, gender, and age. The decline rate of ppFEV1 was higher for children with higher baseline ppFEV1.
Birket et al. [1]	2016	In vitro study	-	Assessed the effects of IVA on airway functional microanatomy and ion transport.	IVA showed beneficial effects over airway functional microanatomy along with decreased mucus viscosity.
Adam et al. [24]	2018	In vitro study	-	Evaluated the Orkambi effect on the repair of primary lung epithelium with PA infection.	The study found that Orkambi showed significant and slight repair of lung epithelium even in the presence of PA infections.

Impact of lumacaftor and Ivacaftor on HRQoL and BMI

 *HRQoL and PEx*

This chronic illness is affecting the HRQoL of patients. The best indicator to reflect the overall health and treatment is via patient-reported outcomes (PROS), so what they feel and what type of treatments benefit their real-life [5]. A valid -disease-based tool is the Cystic Fibrosis Questionnaire-Revised (CFQ-R) that covers the specific areas of HRQoL and those related to the disease. A cross-sectional study by Bell et al., performed in 2019, compared IVA-G551D and F508del-Standard of care (SOC) in the context of HRQoL in patients age >12 years and age six-11 years. They found IVA-G551D showed a better response to HRQoL as compared to F508del-SOC [5].

LUM/IVA is associated with extensive survival years, and therapy results in various ways to affect patients' lives either by decreasing the PEx or hospitalization. Rubin et al. designed a Model Cohort Study in 2000 patients in 2019. They compared the treatment effects in patients with LUM/IVA with SOC and those with SOC only. They displayed that average life expectancy (LE) and additional survival years were promising in LUM/IVA compared with the SOC group [6].

Another Model Cohort study by Dilokthornsakul et al. evaluated the life-time outcomes of LUM/IVA in F508del homozygous patients. They also compared the average LE and quality-adjusted life-years (QALYs). This study concluded the improvement observed in LE and QALYS, corresponding to 27.6% and 20.7%, with the extra life-years as 2.91 life-years and extra QALYS as 2.42 [25].

PEx has a vital role in HRQoL; the more the rate of PEx in the disease course, the greater the chances of hospitalization, Abx use, and deterioration of patients' physical and general health. A qualitative study was published in 2016 by Martin et al. on 26 patients on IVA treatment. It demonstrated the positive impact of IVA on HRQoL. They also found a decreased pattern of PEx, together with a decline rate of Hospital visits and stays [26]. A cross-sectional study on 80 patients by Flume et al. in 2019 evaluated the effects of PEx on HRQoL by using CFQ-R. The result was a score of 8 out of 12 domains of HRQoL in the 1st-week of PEx and took several weeks to recover to reach pre-PEx levels [27].

Physical Activity and Exercise Tolerance

The disease mostly affects the lungs and makes the lung's reserve capacity to the level to which patients cannot do physical activities and exercise. The repeated episodes of a bacterial infection caused the condition worse even to breathe normally. However, LUM/IVA has a well-established role in physical activity and exercise tolerance. A two-years, clinical trial was designed on 03 patients by Savi et al. in 2019. They exhibited the advanced effects of LUM/IVA (pre-and post-initiation) on physical activity and exercise-tolerance [28]. Another study was conducted by Wark et al. on 10 patients. They observed these effects in patients with severe lung disease with FEV1<40 and concluded an improvement at four-week treatment as 78 meters with LUM/IVA in a six-minute walk test (6MWT), which was conserved at 52-week (118.1 m) as well [29].

 *Effects on BMI*

The nutritional status of the patients is directly related to their pancreatic and enzymatic activities. A progressive and chronic illness led them to many nutritional deficiencies by a repeated bacterial infection and unable to gain a normal BMI. The developments of new drugs like LUM and IVA have been giving coverage to this disease area. Tienery et al. performed a prospective study in 2018 over 10 patients. This prospective study assessed the outcome of IVA in G551D patients during five months and 24 months. They observed the consequences of gaining weight and BMI in five-months with a plateau level at 24 months of IVA treatment [30]. Another study, a clinical trial by Lacotuci et al. in 2016, evaluated the IVA effects at six and 12 months of duration. The BMI's clinical impact was displayed as 23.2 kg/m2 from 22 kg/m2 with a better sweat chloride test and reduced PEx rates [31].

Stavely et al. evaluated IVA's role in a linear growth velocity in pediatric patients aged (six-11 years) with G551D. They enrolled 48 patients in the ENVISION study (a placebo-controlled, multicenter Phase 3 IVA trial) with 25-patients on placebo and 23-patients on IVA to assess the height, weight z-score with growth velocities from baseline (BL) to 48-weeks. The placebo group showed a decline in weight with no changes in height z-scores. However, the IVA group reflected the increasing trend in height 0.00 at BL, 0.11 at 24 weeks, P<0.01 and 0.17 at 48 weeks, P<0.001, and weight z-score 0.08 at BL and 0.44 at 48-weeks, P<0.001. The height velocities increased in the IVA-treated group as compared to the placebo group [32].

In their clinical trial, Salvatore et al. had attained improved BMI, CFQ-R, FVC, and FEV1 [33]. Volkova et al. studied the IVA's combined effects in an observational study that compared IVA with a comparator (COMP) group in 2016. They included 9936 participants with IVA in 2014 of the US and UK CF registry. This study yielded a lower rate of PEx, Hospitalization, death rate, and organ-transplantation in the IVA group [34]. A summary of the studies showing an overview of the effects on HRQoL and BMI is shown in Table 2.

**Table 2:** Summary of studies on HRQoL and BMI COMP: comparator; SOC: standard of care; LUM: lumacaftor; IVA: ivacaftor; QALYs: quality-adjusted life-years; CFQ-R: Cystic Fibrosis Questionnaire-Revised; CF: cystic fibrosis; LE: average life expectancy; PEx: pulmonary exacerbations; muts: mutations; BL: baseline; 6MWT: six-minute walk test; FVC: forced vital capacity; FEV1: forced expiratory volume in one second; BMI: basal metabolic index; CFTR: cystic fibrosis transmembrane conductance regulator; HRQoL: health-related quality of life

Author Name	Year of Publication	Type of Study	Number of Patients	Purpose of the Study	Results/Conclusions
Volkova et al. [34]	2016	Observational study	9936	They assessed IVA's safety outcomes in treated patients in 2014 of the US and UK CF registry with the COMP group.	The death rate, Hospitalization, PEx, organ-transplantation are relatively lower in the IVA group as compared to the COMP group that was concluded in the study.
Rubin et al. [6]	2019	Modeling study cohort	2000	Evaluated LUM/IVA+SOC's long-term health effects in patients homozygous for F508del compared to patients with SOC alone.	They found an increased median survival rate, lung function, and delayed lung transplantations among patients on LUM/IVA. They also revealed additional survival with early age drug initiation strategy and treatment continuation.
Dilokthornsakul et al. [25]	2017	Modeling study cohort	1000	Estimated and compared LE and QALYs for age-matched CF patients and non-CF population.	The study observed improvements in LE and QALYS corresponding to 27.6% and 20.7% between CF and non-CF persons for matched age.
Bell et al. [5]	2019	Cross-sectional study	209	The study compared the health outcomes in patients with G551D treated with IVA to the patients with F508del receiving SOC before treatment with LUM/IVA.	Patients with IVA-G551D depicted better response to HRQoL as compared to those with F508del-SOC based on disease-specific measures and genetic mutation in a real-life setting.
Flume et al. [27]	2019	Cross-sectional study	80	They assessed the impact of PEx on the HRQoL of patients using CFQ-R data from two trials in their research.	Scores on the CFQ-R were calculated 8 out of the total 12 domains of HRQoL within the first week of PEx. Several weeks were required for a full recovery to reach pre PEx levels in CF patients.
Stavely et al. [32]	2016	Clinical trial	48	The study assessed IVA's impact on the children's linear growth of weight and height assessment at intervals and compared with the placebo.	Patients enrolled in the ENVISION group showed an increase in height and weight z- score in IVA-treated children compared with the placebo group from BL to 48-weeks. Also, increased growth velocity in the IVA group was observed by them.
Martin et al. [26]	2016	Qualitative study	26	Observed the perceived changes by patients on HRQoL with IVA treatment.	They found that patients aged >12 years and caregivers of six-11 years aged patients reported significant improvement in HRQoL in decreasing PEx and less negative impacts (Hospitalization, clinical visits, the burden of additional treatments).
Tierney et al. [30]	2018	Prospective study	20	Evaluated the clinical implications of IVA on weight and BMI in G551D-patients at five and 24 months of drug initiation.	They reflected a weight and BMI gain in the first five months for IVA treatment with plateau effects for two years.
Lacotucci et al. [31]	2016	Clinical trial	18	Clinical effects of IVA during six months and 12 months of treatment duration.	IVA showed clinical improvement in FEV1, PEx rates, BMI, and sweat chloride test at six and 12 months of treatment.
Wark et al. [29]	2019	Clinical trial-case control	10	They observed the effects of LUM/IVA on lung function and exercise capacity in adults with severe lung disease as FEV1<40%.	The study showed a significant improvement in 6MWT at four-weeks and conserved at 52 weeks in patients with severe airway disease.
Salvatore et al. [33]	2018	Clinical trial	09	They assessed the efficacy and safety of IVA in patients with residual function mutations (muts).	They displayed improved FVC and FEV1 and increased BMI, and good CFQR score in CFTR-residual function muts and severe lung disease.
Savi et al. [28]	2019	Clinical trial	03	Assessed the physical activity in two-year pre- and post-initiation of LUM/IVA.	The clinical trial showed an improved daily physical activity and exercised tolerance after two-year therapy with LUM/IVA.

General impact over other systems of CF patients

Pituitary Gland

This disease is affecting almost all the essential organs of the patients. The pituitary gland is the crucial organ affected by this illness, resulting in Growth hormone deficiency (GHD) [35]. LUM/IVA also covered this defect in terms of causing significant improvement in levels of GH. Pascucci et al., in 2019, conducted a clinical trial over 10 patients, enlisted from previous studies of GHD with Growth hormone-releasing hormone (GHRH) and arginine test, and five patients were LUM/IVA treated [35]. They found that two patients with severe GHD showed a normal response to the Growth hormone-insulin-like growth factor-1 axis test (GH-IGF-1 axis), and two patients with partial deficiency showed normal test response.

Anemia

The malabsorption in the gut in these patients is responsible for losing essential nutrients necessary for their average growth. And like other chronic ailments, anemia is the prominent factor affecting the general well-being of an individual. One of the studies, by Gifford et al. in 2019, testified the concept of LUM/IVA impact on the rise of hemoglobin (Hgb) level [36]. In a registry-based study of 13929 CF patients, they concluded that IVA increases 0.54 gm/dL in males and 0.18 gm/dL in females, and LUM/IVA increased 0.58gm/dL in males and 0.26 gm/dL in females.

Pregnancy/Fertility and Fetus Exposure

Pregnancy was a challenging milestone before the introduction of CFTR modulators. The ailment coverage over the reproductive system and cervical mucous viscosity imposed them on subfertility and infertility. In 2019, a review article by Jennifer et al. summarized the overall efficacy of CFTR modulators in pregnancy, fertility, and lactation. IVA treatment resulted in pregnancy, even in females using contraceptives during the study duration, as treatment increased the CFTR expression and general health benefits. They didn't find specific studies on the CFTR modulator's effects on neonatal exposure in this review [37]. The study displayed evidence of no congenital anomalies reported in many case reports with women treated with IVA in all three trimesters, with only a few cases showed premature birth in these pregnant ladies with compromised lung capacity. The effects of LUM/IVA in lactation exposed a slight variation in liver function test with a healthy eye examination of neonates.

Trimble et al. reported a study of a pregnant lady on LUM/IVA treatment during her pregnancy, who delivered a healthy baby and successfully breastfed her baby with no significant elevations in liver function tests were observed [38]. However, they suggested further data for the safety of these drugs in pregnancy and breastfeeding. Table 3 shows studies related to the general impact over other systems.

**Table 3:** Summary of studies of general impact over other systems LUM: lumacaftor; IVA: ivacaftor; Hbg: hemoglobin; CF: cystic fibrosis; GHD: growth hormone deficiency; GH-IGF-1 axis: growth hormone-insulin-like growth factor-1 axis; CFTR: cystic fibrosis transmembrane conductance regulator

Author Name	Year of Publication	Type of Study	Number of Patients	Purpose of the Study	Results/Conclusions
Gifford et al. [36]	2019	Model study	13929	Evaluated the effects of LUM/IVA on Hgb levels in G551D and F508del patients.	The study showed an average increase in Hbg levels with the treatment of LUM/IVA.
Pascucci et al. [35]	2019	Clinical trial	10	Assessed LUM and IVA's effect on the patients, from previous studies, on damaged GH-IGF-1 axis.	LUM/IVA showed significant correction in GHD.
Jennifer et al. [37]	2019	Review article	-	They reviewed the efficacy of CFTR modulators in pregnancy and fertility.	They reported fertility and successful pregnancies in females with the CFTR modulators.
Trimble et al. [38]	2018	In vitro Study	01	The study reflected the impact of LUM/IVA on one pregnant lady and breastfeeding.	They reported delivery of health baby in mother of CF patient and no significant liver function test elevations on breastfeeding.

## Conclusions

This review article focused on the clinical efficacy of LUM/IVA in CF patients. It covers all the fields of interest regarding better impact over lung functions, PEx, Hospitalization rates, HRQoL, physical activity, exercise tolerance, and BMI. LUM/IVA combination resulted in a declined mortality rate by reducing the episodes of severe lung infections and making the life of these patients comfortable with the achievement of adequate pulmonary function without further worsening along with reduced Hospital visits. The hopeful impact of treatment on lung tissue repair is making the lung condition better. The early initiation of these drugs gives long-term benefits to patients of all age groups and premature discontinuation, resulting in a relapse of their disease. The better HRQoL makes them participate and continue their everyday life activities like school, college, office, household activities. The improved growth velocities rates among the pre-pubertal age with LUM/IVA help them achieve BMI close to their expected years. Patients getting ordinary SOC instead of LUM/IVA faced many complications related to their disease and disease progression. The essential findings of progressed lung functions are positively affecting their general health.

Despite all the studies that we collected and reviewed, more studies on the clinical effects of LUM/IVA in all age groups are necessary to be conducted by others on a large number of the study sample for better and reliable results. There are fewer data available on LUM/IVA safety profile in pregnancy, fertility, and breastfeeding; additional studies are necessary to look for drug safety and efficacy.

## References

[1] Birket SE, Chu KK, Houser GH (2016). Combination therapy with cystic fibrosis transmembrane conductance regulator modulators augment the airway functional microanatomy. Am J Physiol Lung Cell Mol Physiol.

[2] Feng LB, Grosse SD, Green RF, Fink AK, Sawicki GS (2018). Precision medicine in action: the impact of ivacaftor on cystic fibrosis-related hospitalizations. Health Aff (Millwood).

[3] Heltshe SL, Cogen J, Ramos KJ, Goss CH (2017). Cystic fibrosis: the dawn of a new therapeutic era. Am J Respir Crit Care Med.

[4] Zhang W, Zhang X, Zhang YH, Strokes DC, Naren AP (2016). Lumacaftor/ivacaftor combination for cystic fibrosis patients homozygous for Phe508del-CFTR. Drugs Today (Barc).

[5] Bell SC, Mainz JG, MacGregor G (2019). Patient-reported outcomes in patients with cystic fibrosis with a G551D mutation on ivacaftor treatment: results from a cross-sectional study. BMC Pulm Med.

[6] Rubin JL, O'Callaghan L, Pelligra C (2019). Modeling long-term health outcomes of patients with cystic fibrosis homozygous for F508del-CFTR treated with lumacaftor/ivacaftor. Ther Adv Respir Dis.

[7] Tesell MA, Alper CJ, Bacon R (2019). Effect of lumacaftor/ivacaftor on pulmonary exacerbation rates in members with cystic fibrosis in a medicaid population. J Manag Care Spec Pharm.

[8] Wu YS, Jiang J, Ahmadi S (2019). ORKAMBI-mediated rescue of mucociliary clearance in cystic fibrosis primary respiratory cultures is enhanced by arginine uptake, arginase inhibition, and promotion of nitric oxide signaling to the cystic fibrosis transmembrane conductance regulator channel. Mol Pharmacol.

[9] Burgel PR, Munck A, Durieu I (2020). Real-life safety and effectiveness of lumacaftor-ivacaftor in patients with cystic fibrosis. Am J Respir Crit Care Med.

[10] Mayer-Hamblett N, Boyle M, VanDevanter D (2016). Advancing clinical development pathways for new CFTR modulators in cystic fibrosis. Thorax.

[11] Brodsky JL, Frizzell RA (2015). A combination therapy for cystic fibrosis. Cell.

[12] McColley SA, Konstan MW, Ramsey BW (2019). Lumacaftor/Ivacaftor reduces pulmonary exacerbations in patients irrespective of initial changes in FEV1. J Cyst Fibros.

[13] Konstan MW, McKone EF, Moss RB (2017). Assessment of safety and efficacy of long-term treatment with combination lumacaftor and ivacaftor therapy in patients with cystic fibrosis homozygous for the F508del-CFTR mutation (PROGRESS): a phase 3, extension study. Lancet Respir Med.

[14] Diab-Cáceres L, Girón-Moreno RM, Pastor-Sanz MT (2018). Compassionate use of lumacaftor/ivacaftor in cystic fibrosis: Spanish experience. Arch Bronconeumol.

[15] Hassan M, Bonafede MM, Limone BL, Hodgkins P, Suthoff ED, Sawicki G (2016). 28 Reduction in pulmonary exacerbations (PEx) after initiation of ivacaftor: a retrospective cohort study among patients with cystic fibrosis (CF) treated in real-world settings. J Cyst Fibros.

[16] Labaste A, Ohlmann C, Mainguy C (2017). Real-life acute lung function changes after lumacaftor/ivacaftor first administration in pediatric patients with cystic fibrosis. J Cyst Fibros.

[17] Burgel PR, Durieu I, Chiron R (2020). Clinical response to lumacaftor-ivacaftor in patients with cystic fibrosis according to baseline lung function. J Cyst Fibros.

[18] Tong K, Barker D, France M (2020). Lumacaftor/ivacaftor reduces exacerbations in adults homozygous for Phe508del mutation with severe lung disease. J Cyst Fibros.

[19] Grady EO, Finnegan R, Smyth A (2019). P051 The bigger you are, the harder you fall? Short term effects of LUM/IVA (Orkambi) on lung function in children with cystic fibrosis. Arch Dis Child.

[20] Zhang S, Shrestha CL, Kopp BT (2018). Cystic fibrosis transmembrane conductance regulator (CFTR) modulators have differential effects on cystic fibrosis macrophage function. Sci Rep.

[21] Barnaby R, Koeppen K, Nymon A (2018). Lumacaftor (VX-809) restores the ability of CF macrophages to phagocytose and kill Pseudomonas aeruginosa. Am J Physiol Lung Cell Mol Physiol.

[22] Singh SB, McLearn-Montz AJ, Milavetz F (2019). Pathogen acquisition in patients with cystic fibrosis receiving ivacaftor or lumacaftor/ivacaftor. Pediatr Pulmonol.

[23] Schneider EK, Azad MA, Han ML (2016). An "unlikely" pair: the antimicrobial synergy of polymyxin B in combination with the cystic fibrosis transmembrane conductance regulator drugs KALYDECO and ORKAMBI. ACS Infect Dis.

[24] Adam D, Bilodeau C, Sognigbé L, Maillé É, Ruffin M, Brochiero E (2018). CFTR rescue with VX-809 and VX-770 favors the repair of primary airway epithelial cell cultures from patients with class II mutations in the presence of Pseudomonas aeruginosa exoproducts. J Cyst Fibros.

[25] Dilokthornsakul P, Patidar M, Campbell JD (2017). Forecasting the long-term clinical and economic outcomes of lumacaftor/ivacaftor in cystic fibrosis patients with homozygous phe508del mutation. Value Health.

[26] Martin Mona, Mccarrier KP, Hassan M (2016). 27 Impact of treatment with ivacaftor (IVA) among patients with cystic fibrosis (CF): a qualitative study to evaluate patient-perceived changes in aspects of daily life. J Cyst Fibros.

[27] Flume PA, Suthoff ED, Kosinski M, Marigowda G, Quittner AL (2019 Sep). Measuring recovery in health-related quality of life during and after pulmonary exacerbations in patients with cystic fibrosis. J Cyst Fibros.

[28] Savi D, Schiavetto S, Simmonds NJ, Righelli D, Palange P (2019). Effects of Lumacaftor/Ivacaftor on physical activity and exercise tolerance in three adults with cystic fibrosis. J Cyst Fibros.

[29] Wark PAB, Cookson K, Thiruchelvam T, Brannan J, Dorahy DJ (2019). Lumacaftor/ Ivacaftor improves exercise tolerance in patients with cystic fibrosis and severe airflow obstruction. BMC Pulm Med.

[30] Tierney A, King SJ, Edgeworth D (2018). P204 An increase in weight and fat mass observed following five months of ivacaftor treatment plateaus at 24 months in adults with G551D-related cystic fibrosis. J Cyst Fibros.

[31] Lacotucci P, Salvatore D, Carnovale V (2016). 30 Effects of ivacaftor in cystic fibrosis patients carrying a non-G551D gating mutation with severe lung disease. J Cyst Fibros.

[32] Stalvey MS, Niknian M, Higgins M, Tarn V, Heltshe SL, Rowe SM (2016). 197 Ivacaftor improves linear growth in children with cystic fibrosis (CF) and a G551D-CTFR mutation: data from the ENVISION study. J Cyst Fibros.

[33] Salvatore D, Braggion C, Messore B, Pisi G, Tuccio G, Bena C, Colangelo C (2018). IPD2.05 Effects of ivacaftor in patients with cystic fibrosis and severe lung disease-carrying CFTR mutations with residual function. J Cyst Fibros.

[34] Volkova N, Bai Y, Higgins M, Bengtsson L, Tian S, Nyangoma S, Bilton D (2016). ePS03.1 Disease progression in patients (pts) with cystic fibrosis (CF) treated with ivacaftor (IVA): analysis of real-world data from the UK CF registry. J Cyst Fibros.

[35] Pascucci C, De Biase RV, Savi D (2019). Impact of CFTR-modulating drugs on GH-IGF-1 axis impairment in adult patients with cystic fibrosis. J Endocrinol Invest.

[36] Gifford AH, Heltshe SL, Goss CH (2019). CFTR modulator use is associated with higher hemoglobin levels in individuals with cystic fibrosis. Ann Am Thorac Soc.

[37] Taylor-Cousar JL (2020). CFTR modulators: impact on fertility, pregnancy, and lactation in women with cystic fibrosis. J Clin Med.

[38] Trimble A, McKinzie C, Terrell M, Stringer E, Esther CR Jr (2018). Measured fetal and neonatal exposure to Lumacaftor and Ivacaftor during Pregnancy and while breastfeeding. J Cyst Fibros.

